# Blue angels have devil hands: Predatory behavior using cerata in *Glaucus atlanticus*

**DOI:** 10.1002/ecy.70062

**Published:** 2025-03-17

**Authors:** Gaku Yamamoto, Naoki Kanai, Toru Miura, Kohei Oguchi

**Affiliations:** ^1^ Enoshima Aquarium Fujisawa Kanagawa Japan; ^2^ Ocean Research Explorations Honolulu Hawaii USA; ^3^ Misaki Marine Biological Station, The University of Tokyo Miura Kanagawa Japan; ^4^ Undergraduate School of Biological Sciences, Department of Biology Tokai University Sapporo Hokkaido Japan

**Keywords:** cerata, *Glaucus*, kleptocnidae, nudibranch, pleuston, predatory behavior

Nudibranchs, a subset of gastropods within the phylum Mollusca, encompass over 3000 valid species worldwide, characterized by the thinning or internalization of a shell (Do et al., 2022; Goodheart et al., 2015; Valdés, 2004). In place of shells, nudibranchs have evolved various alternative defense tactics, including vibrant warning or camouflage coloration (Paul & Ritson‐Williams, 2008; Wägele & Klussmann‐Kolb, 2005). Among nudibranch species belonging to Cladobranchia, most employ nematocysts stolen from dietary benthic cnidarians such as hydrozoans and anemones for defense, as known as “kleptocnidae.” These nematocysts are incorporated inside dorsal projections called “cerata (singular: ceras)” (Edmunds, 1966; Goodheart et al., 2017, 2018; Greenwood, 2009; Grosvenor, 1903; Kepner, 1943; Putz et al., 2010). Nematocysts are a type of organelle unique to cnidarians; these pouch‐like structures invert in response to mechanical and/or chemical stimuli, to release toxic needles (Holstein & Tardent, 1984). Remarkably, cladobranchs can capture and store nematocysts in the distal part of each ceras in an organ called the “cnidosac,” which are expelled when attacked by predators (Goodheart et al., 2017, 2018; Greenwood, 2009; Grosvenor, 1903). During the process of incorporating the ingested nematocysts, they are transported through the digestive tract into specialized cells called “cnidophages” located in the cnidosac (Goodheart et al., 2017, 2018; Greenwood, 2009; Grosvenor, 1903).

Unlike most cladobranchs, which have cerata on their dorsal sides, all species of the genus *Glaucus* (the sole genus in the family Glaucidae), bear several paired fin‐like projections on each side of their bodies (Thompson & Bennett, 1970; Thompson & McFarlane, 1967). *Glaucus* species live by floating with air inside their bodies and their ventral side facing the surface of the water (Thompson & McFarlane, 1967). Due to their distinctive body plan and their silvery‐white dorsal and blue ventral coloration, they are often called “blue angels,” “blue dragons” or “sea swallows” (Figure 1a). Unlike many other nudibranchs which are benthic, all species of *Glaucus* are pleuston (sometimes termed neuston) species that live on the ocean's surface, using cerata and air bubbles in their stomach cavities for buoyancy (Miller, 1974; Thompson & Bennett, 1970; Thompson & McFarlane, 1967). They are carnivorous and prey on other pleustonic cnidarian species, including bluebottles (*Physalia* sp.), sea rafts (*Velella velella*), and blue buttons (*Porpita porpita*) (Bieri, 1966; Helm, 2021; Figure 1b,c; see Video_S1.mov, Video_S2.mov, Video_S3.mov in Oguchi 2024). Similar to other cladobranchs, *Glaucus* species engage in kleptocnidae, recycling nematocysts from cnidarians, likely for defense against predators (Thompson & Bennett, 1970; Valdés & Campillo, 2004). Because of the difficulty of rearing *Glaucus* species, together with the unclear ecological relationships among pleuston species, the function and adaptive significance of cerata and kleptocnidae in *Glaucus* are poorly understood. Here, we report that *Glaucus atlanticus* successfully reared in aquaria employ their cerata for prey capture, suggesting that the role of kleptocnidae is not limited solely to defense.

**FIGURE 1:**
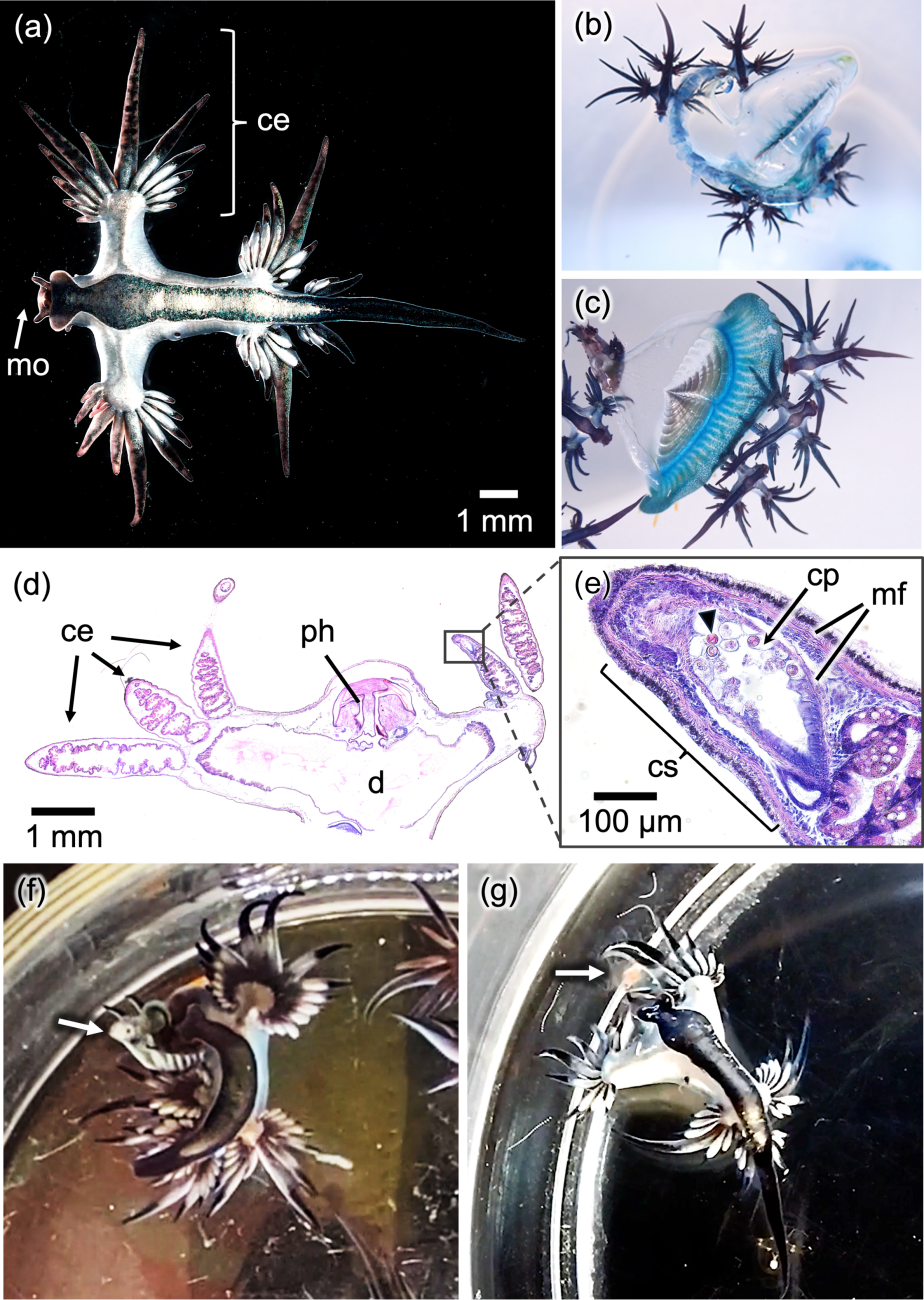
Morphological, behavioral, and histological features of *Glaucus atlanticus*. Ventral view of whole‐body image of *G. atlanticus* (a). Predatory behavior toward *Physalia utriculus* (b) and *Velella velella* (c). Transverse histological section of an individual (d) and enlarged images of the distal part of a ceras (e) stained with hematoxylin and eosin. Predatory behavior with cerata toward live whitebait *Engraulis japonicus* (f) and jellyfish *Rathkea octopunctata* (g). Arrowhead: Nematocysts; mo: Mouth; ce: Cerata; ph: Pharynx; d: Digestive tract; cp: Cnidophage; cs: Cnidosac; mf: Muscle fibers. White arrow indicates whitebait (f) or jellyfish (g) respectively. Photographs: Gaku Yamamoto (a–c, f, g); Naoki Kanai (d, e).

Specimens of *G. atlanticus* were collected on June 10, 2020, and August 16, 2023, at Katase Nishihama Beach (35°18′48.6″ N, 139°28′23.3″ E) and February 20, 2024, at Araihama Beach (35°09′33.0″ N, 139°36′42.8″ E) in Japan. *Physalia* nematocysts were incorporated into the cnidosac and cnidophage at the cerata tips in *G. atlanticus* (Goodheart et al., 2018; Figure 1d,e; Appendix S1: Figure S1). When some specimens of *G. atlanticus* were reared with live whitebait, that is, *Engraulis japonicus* juveniles, they surprisingly employed the most anterior cerata as “hands” to capture whitebait to consume it (Figure 1f). Immediately after whitebait were introduced, *G. atlanticus* was observed to actively direct its cerata toward the fish (Appendix S1: Figure S2A; see Video_S4.mov in Oguchi, 2024). Subsequently, upon contact between the cerata and the whitebait, *G. atlanticus* twisted its body, actively aiming its mouth at the whitebait in an attempt to consume it. During continuous behavioral observations, *G. atlanticus* held the whitebait between the cerata and began biting near the gills of the whitebait (Appendix S1: Figure S2A; see Video_S4.mov in Oguchi, 2024). Approximately 20 min after capture, the whitebait was consumed from the gill area to the tail, with only the head remaining (Appendix S1: Figure S2A; see Video_S4.mov in Oguchi, 2024). Furthermore, when provided with thawed frozen whitebait, all *G. atlanticus* individuals used their cerata to grasp the fish ventrally, rolling into a dorsal‐side‐up position (see Video_S5.mov in Oguchi, 2024). In addition to fish, various jellyfish species, including comb jellies (Ctenophora), were provided to *G. atlanticus* for predation tests (Table 1; see Video_S6.mov, Video_S7.mov, Video_S8.mov, Video_S9.mov in Oguchi, 2024). The results revealed that *G. atlanticus* preyed on all tested cnidarian jellyfish, including known hydrozoan prey such as *Physalia* and *Porpita*, as well as species newly found to be prey (Figure 1g; Appendix S1: Figure S2B; Table 1). The predatory use of the most anterior cerata as “hands” was also observed during cnidarian predation (see Video_S6.mov and Video_S7.mov in Oguchi, 2024). In contrast, no similar predatory behavior was observed with comb jellies (Table 1; see Video_S8.mov and Video_S9.mov in Oguchi, 2024).

**TABLE 1:** List of animals provided to, and the success of predation by, *Glaucus atlanticus*.

Phylum	Class	Order	Species	Predatory behavior
Cnidaria	Hydrozoa	Siphonophore	*Physalia utriculus*	Observed
		Anthoathecata	*Porpita porpita*	Observed
			*Velella velella*	Observed
			*Rathkea octopunctata*	Observed
		Leptothecata	*Eutonina indicans*	Observed
			*Clytia* sp.	Observed
	Scyphozoa	Semaeostomeae	*Aurelia coerulea*	Observed
Ctenophora	Tentaculata	Lobata	*Bolinopsis mikado*	Not observed
	Nuda	Beroida	*Beroe cucumis*	Not observed
			*Beroe campana*	Not observed
Vertebrate	Actinopterygii	Clupeiformes	*Engraulis japonicus*	Observed

Additionally, *Glaucus marginata* exhibited a comparable series of predatory behaviors toward fish and several cnidarians (see Video_S3.mov in Oguchi, 2024). Most Cladobranchia species are known to be specialists, feeding exclusively on specific cnidarians (Goodheart et al., 2017). However, our observations demonstrate that *Glaucus* not only feeds on pleustonic cnidarians, but also has a broader diet, capturing various cnidarian species and fish for their food, suggesting a wider feeding spectrum compared with those of other Cladobranchia species (Table 1).

It has long been postulated that kleptocnidae and cerata in Cladobranchia are mainly defensive organs (Goodheart & Bely, 2017; Putz et al., 2010). As it adapted to sea surface habitats, *Glaucus* shifted cerata positions from the dorsal side to the lateral side of the body and potentially altered the functions of the anteriormost cerata to include use for predation as well as defense. Furthermore, the basal part of the anteriormost cerata is longer than that of the other cerata and may function like an arm. Indeed, the basal portion of the anteriormost cerata was observed to move flexibly to grasp the prey (Figure 1f,g; see Video_S4.mov, Video_S5.mov, Video_S6.mov, Video_S7.mov in Oguchi, 2024). Similarly, several neural mechanisms for flexible movement of cerata in response to external stimuli have been reported in *Berghia stephanieae* (Brown et al., 2024). Thus, specialized development of muscles and motor neuronal circuits in these cerata and their basal portion may enable such skillful movements. Actually, a well‐developed muscle fiber layer was observed under the epithelial layers of cerata and the base part of cerata (Figure 1d,e).

During the evolution of *Glaucus*, dietary habits may have shifted with the acquisition of pleustonic life. Most species of Cladobranchia are benthic and are known to obtain their nematocysts by specialized predation on benthic cnidarians (Anthony et al., 2024; Goodheart et al., 2017, 2018). *Glaucus* is a derived group of Cladobranchia and is the only pleuston species (Anthony et al., 2024; Goodheart et al., 2018). The distributions of pleuston species, including those of *Glaucus*, are not stable, being influenced by ocean currents and winds, so the strategy of specialists that only eat specific species may not have been adaptive. Furthermore, the skillful movements of cerata may have allowed *Glaucus* to capture fast‐moving animals such as whitebait and jellyfish. Consequently, as revealed in this study, *Glaucus* may have evolved to prey on a wide variety of organisms, not just cnidarians. In recent years, DNA metabarcoding analysis through high‐throughput amplicon sequencing has emerged as a comprehensive method for investigating stomach contents, providing information on diet repertoires (e.g., Damian‐Serrano et al., 2022; Hetherington et al., 2022; Pringle & Hutchinson, 2020). For example, metagenomic analysis of the stomach contents of the pleuston species *P. physalis* revealed that it preys on a greater variety of animal taxa than other planktonic species of the same group (Cystonectae) (Damian‐Serrano et al., 2022; Hetherington et al., 2022). Employing similar approaches to study the gut contents of *Glaucus* in its natural habitat may reveal its actual dietary preferences. Our findings shed light on hidden food webs and could provide new insights into the ecology and evolution of the marine surface layer.

Overall, our observations reveal that *Glaucus* nudibranchs (1) consume venomous jellyfish, such as *Physalia*, incorporating their nematocysts into the cnidophages of the cerata, (2) use their anteriormost cerata as functional hand‐like appendages for predation, and (3) demonstrate generalist rather than specialist feeding, consuming various cnidarian species and even fish.

## AUTHOR CONTRIBUTIONS

Gaku Yamamoto and Kohei Oguchi conceptualized and designed the study. Gaku Yamamoto conducted rearing and behavioral observations of the *Glaucus*. Naoki Kanai, Kohei Oguchi, and Toru Miura performed histological observations. All authors wrote the manuscript and approved the final version of the manuscript.

## CONFLICT OF INTEREST STATEMENT

The authors declare no conflicts of interest.

## Supporting information


Appendix S1:

## Data Availability

Original videos (Oguchi, 2024) are available in Figshare at https://doi.org/10.6084/m9.figshare.26403493.v1.
