# Supplementary material for: Blue angels have devil hands: Predatory behavior using cerata in Glaucus atlanticus
Source: Ecology. 2025 Mar 17;106(3):e70062. doi: 10.1002/ecy.70062 (PMC11912302; doi:10.1002/ecy.70062)
Supplement: Supplementary file 1 — Appendix S1: [file ECY-106-e70062-s001.pdf]

Appendix S1

Supplementary information for:

Blue angels have devil hands: Predatory behavior using cerata in *Glaucus atlanticus*

Gaku Yamamoto, Naoki Kanai, Toru Miura, Kohei Oguchi

Ecology

This file includes:

Supplementary Figures and Captions (Figures S1 and S2)

Supplementary Figures and Captions

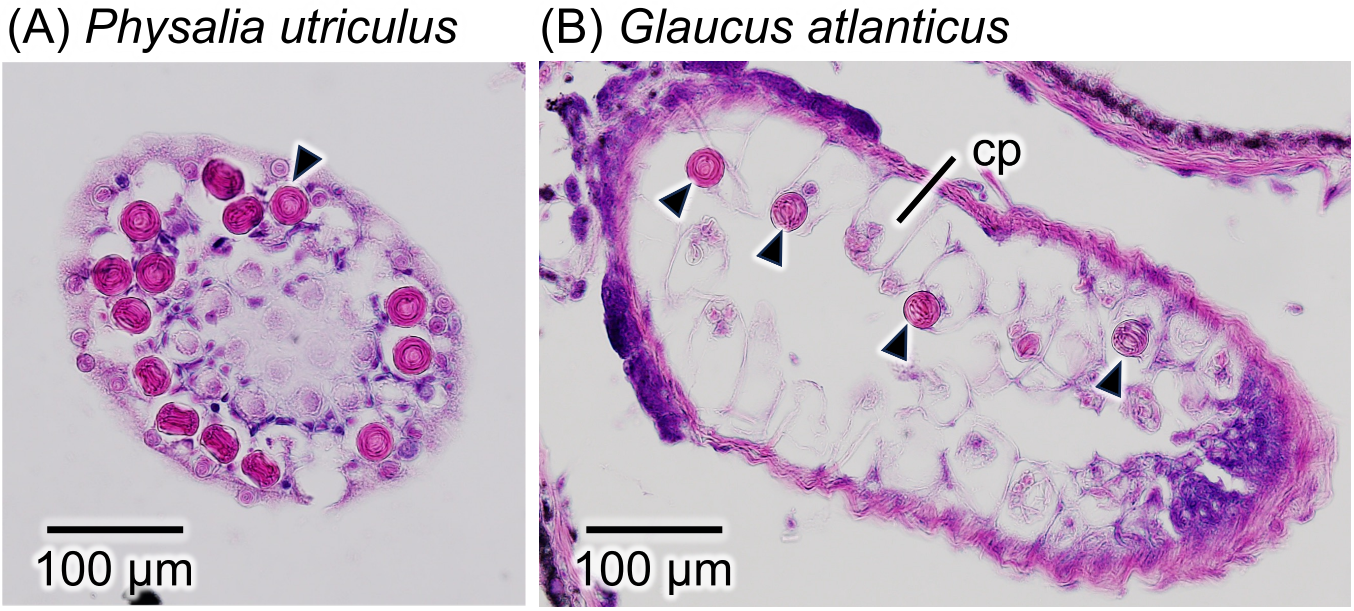

Figure S1.

Characteristics of nematocysts in *Physalia utriculus* and *Glaucus atlanticus*. Transverse histological section of *P. utriculus* tentacles (**A**). An enlarged image of the cnidosac in *G. atlanticus* (**B**). Both images exhibit similar nematocyst morphology and strong staining with eosin. Arrowhead: nematocytes; cp: cnidophage. Photographs: Naoki Kanai.

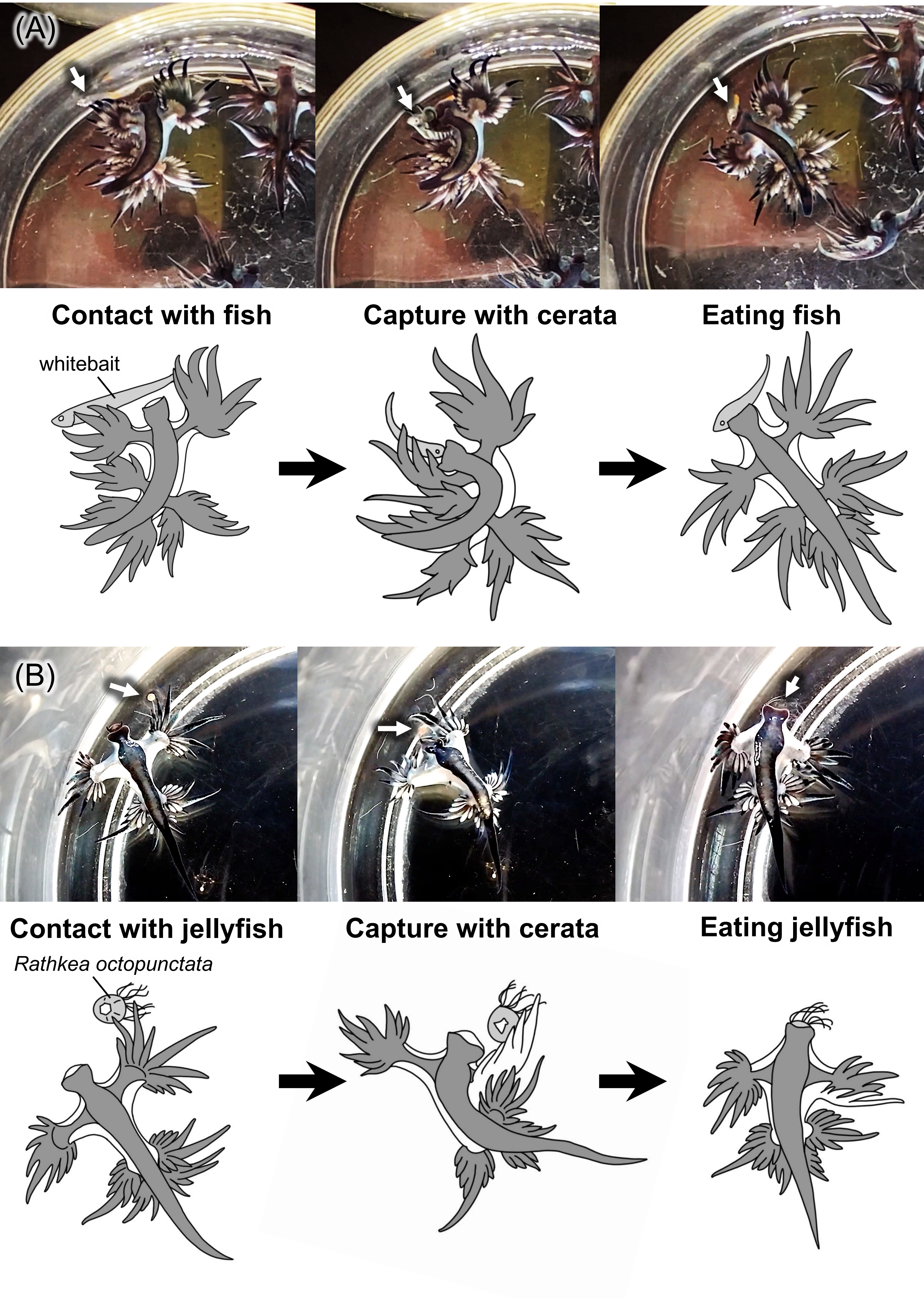
(A)
Contact with fish
Capture with cerata
Eating fish
whitebait
(B)
Contact with jellyfish
Capture with cerata
Eating jellyfish
Rathkea octopunctata

Figure S2.

Sequence of predatory behavior with cerata captured from Video_S4.mov and Video_S6.mov in Oguchi (2024) at https://doi.org/10.6084/m9.figshare.26403493.v1. Predatory behavior with cerata toward live whitebait *Engraulis japonicus* (**A**) and jellyfish *Rathkea octopunctata* (**B**). Initial contact of cerata with the whitebait or jellyfish was followed by using cerata to move the prey to the mouth and consume it. Arrow indicates whitebait or jellyfish, respectively. Photographs and illustrations: Gaku Yamamoto.

Reference:

Oguchi, K. 2024. "Videos on predatory behaviour of Glaucus.mov." Figshare, Media. https://doi.org/10.6084/m9.figshare.26403493.v1
